# Recent Advances in Image-Guided Tissue Sampling

**DOI:** 10.7759/cureus.71613

**Published:** 2024-10-16

**Authors:** Talal Musaddaq, Besma Musaddaq

**Affiliations:** 1 Radiology, Watford General Hospital, Watford, GBR; 2 Medicine, University of Cambridge, Cambridge, GBR; 3 Radiology, Royal Free Hospital, London, GBR

**Keywords:** 1. radiology, general radiology, radiology, radiology & imaging, radiology review articles

## Abstract

Recent advances in image-guided tissue sampling have enhanced diagnostic medicine, particularly in oncology. Traditional techniques, such as computed tomography (CT)-, ultrasound (US)-, and magnetic resonance imaging (MRI)-guided biopsies, remain the cornerstone of diagnostic interventions, each offering unique advantages based on tissue characteristics. CT-guided biopsies excel in deeper complex lesions, while US-guided biopsies provide real-time imaging ideal for superficial tissues. MRI-guided biopsies are invaluable for soft tissue evaluations. The emergence of fusion imaging, which combines modalities such as positron emission tomography (PET)/CT or MRI/US, has demonstrated enhanced diagnostic accuracy. Despite these advantages, image co-registration and cost are the main drawbacks. Emerging techniques such as molecular breast imaging (MBI) and shear wave elastography (SWE) have been evaluated, particularly for breast cancer; however, research suggests that US is likely to remain the most effective modality due to both its cost and ease of use. Innovations in biopsy navigation, including augmented reality, “hot needles,” and robotic assistance, demonstrate promise in closing the gap between operator dependency and procedural consistency; however, further research is required. While liquid biopsies show promise in non-invasive early cancer detection, they are not yet ready to replace tissue biopsies. Collectively, these advancements indicate a future where image-guided tissue sampling is more targeted, less invasive, and diagnostically accurate, although cost and technology access remain challenges.

## Introduction and background

The need for identification and targeting of tumours has increasingly grown to be one of the most important areas of medicine. This had led to the growth and advancement of radiology techniques. Over time, these techniques have iteratively become more accurate, multifunctional and provided increased ease to patients. Biopsies are essential to both diagnosis and treatment of oncological pathologies. Existing measures have provided excellent ways of diagnosing and treating patients; however, success rates can be limited depending on the technique. Further advances are being made to help improve patient comfort, diagnostic accuracy, and treatment.

Several techniques are currently being employed in healthcare settings to identify, biopsy, and treat cancers. The most common image-guided biopsy methods are computed tomography (CT)- and ultrasound (US)-guided biopsies. CT guidance is particularly good for deeper tissue biopsies and higher accuracy. US-guided biopsies are typically better for softer tissues that are relatively superficial. For example, typically biopsies located near the bones are conducted with the aid of CT guidance; however, breast biopsies are performed under the guidance of US.

In addition to CT- and US-guided biopsies, other imaging modalities, such as magnetic resonance imaging (MRI)-guided biopsies, are used. MRI-guided biopsy indications include non-palpable breast lesions as well as central nervous system (CNS) lesions, which can be better visualized with MRI. This review explores the effectiveness of traditional biopsy methods and compares them to more recent advances in image-guided tissue sampling.

This study undertook a comprehensive literature review to examine conventional image-guided biopsy methods and recent advancements. The primary objective was to evaluate the diagnostic accuracy, safety, and practical applications of various image-guided techniques in different clinical settings.

## Review

Methods

To achieve this, data from academic databases such as PubMed, Google Scholar, and Medline were used. Keywords used in the search included terms such as "CT-guided biopsy accuracy," "US-guided biopsy complications," "fusion imaging in biopsies," "MRI/CT fusion biopsy," "PET/CT biopsy diagnostic accuracy," "PET/CT US fusion-guided biopsy," "novel image-guided biopsy techniques," "fusion-guided biopsy," "SWE diagnostic accuracy," "augmented reality biopsies," "molecular breast imaging biopsy diagnostic accuracy," "robot-assisted biopsies," and "liquid biopsies." Inclusion criteria focused on studies that provided clear distinctions in outcomes related to different biopsy techniques and advancements, while excluding those lacking sufficient data on diagnostic outcomes or clinical application.

Conventional image-guided techniques

CT-Guided Biopsies

Studies have shown that CT-guided biopsy success rates and corresponding complications differ depending on the tissue being sampled. A CT-guided liver biopsy is a valuable diagnostic tool with high accuracy for evaluating liver lesions. Studies have shown that CT-guided liver biopsy has a diagnostic accuracy ranging from 80% to 95% [1,2]. Complication rates associated with CT-guided liver biopsy are generally low, with reported rates of major complications ranging from 0.6% to 3.2% [3]. Common complications include bleeding, pneumothorax, and infection. Factors that can affect the diagnostic accuracy of CT-guided liver biopsy include lesion size, location, and operator experience.

**Figure 1:**
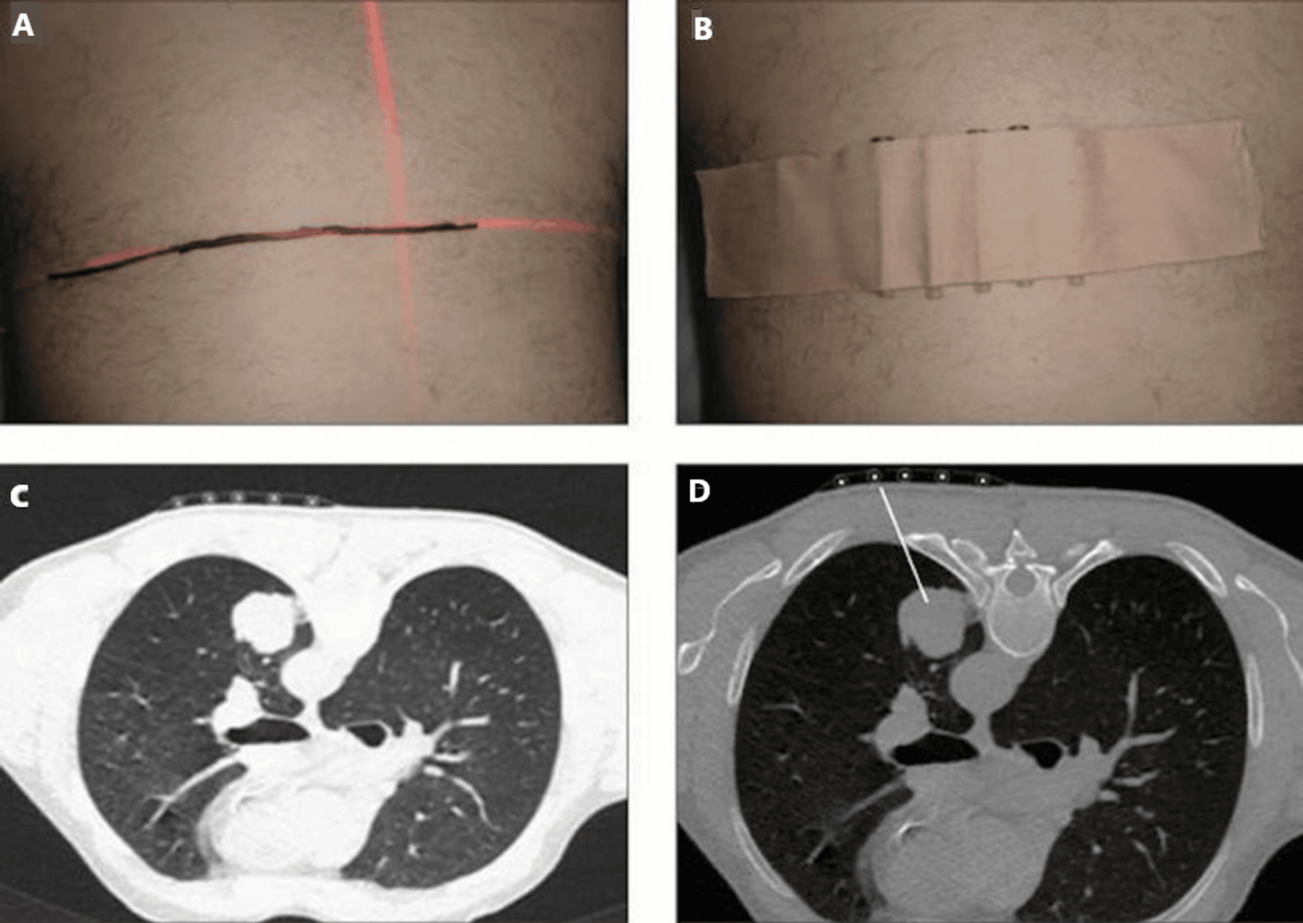
CT-guided lung biopsy process. The lung lesion is identified and marked on the patient's chest, as shown in (A). Radiopaque markers can be applied to increase the accuracy of biopsy, as seen in (B), although not always necessary. A scan is taken again following the application of the radiopaque marker (C), and the shortest distance path determined is determined. The needle is then inserted along that path to biopsy the target lesion (D).

CT-guided lung biopsies are frequently used to investigate lung lesions (Figure 1). According to various studies, CT-guided lung biopsies have a diagnostic accuracy of between 71% and 95%. Pneumothorax is the most common complication, varying between 17% and 26% [4]. According to a review of CT-guided lung biopsies, the technical success rate was 87.2%. The technical success rate was defined in the review as the percentage of biopsy procedures that lead to the acquisition of adequate tissue samples for diagnostic purposes without major technical problems [5]. Spinnato et al. [6], who analysed 2027 CT-guided bone biopsies, concluded that the diagnostic accuracy of CT-guided bone biopsies lies between 90% and 94%. Another study stated that the range was closer to 58%-90% [7]. The difference in the aforementioned ranges is likely driven by differences in operator skill and type of lesion being biopsied. Typically, CT-guided bone biopsies have higher diagnostic accuracy compared to lung CT-guided biopsies due to the ability to visualize bone lesions in detail and access them accurately for biopsies [8]. By contrast, the lung lesions are frequently located deep in the lung parenchyma, and additionally, lung movements may make performing accurate sampling more challenging [9].

US-Guided Biopsies

CT imaging provides detailed visualization of lung lesions, allowing for precise targeting of the biopsy needle. This technique is preferred when the lesions are small and located in deeper, challenging anatomical locations. However, US-guided lung biopsies may be performed in specific situations [10]. For example, US-guided biopsies may be preferred when lung lesions are located near the chest wall or in the pleural space, where they can be easily visualized by US. US-guided biopsies can provide real-time imaging guidance and can be particularly useful for lesions that are superficial and accessible. Additionally, US-guided biopsies may be preferred in cases where there is a concern for tumour seeding, as they have been associated with a lower risk of tumour seeding compared to CT-guided biopsies [11]. Thus, the choice between US-guided and CT-guided lung biopsies depends primarily on the biopsy location and the risk of complications. Diagnostic accuracy for US-guided lung biopsies has been reported between 72% and 87% compared to CT-guided lung biopsies, which are between 71% and 95% [1,12].

Given that US-guided biopsies are more suited for relatively more superficial biopsies, soft tissue sarcomas have been commonly biopsied under US guidance. According to Cernakova et al. [13], US-guided biopsies of soft tissue sarcoma yielded a diagnostic accuracy of 87%-93%. While complications were low in the cohort, two patients with risk factors (cancer, recent surgery, smoking, and venous insufficiency) developed pulmonary emboli several days following the US-guided biopsy. A common complication of soft tissue sarcoma biopsies is the risk of seeding through the inadvertent creation of biopsy seeding tracts, which are more common in open biopsy procedures [14]. It is uncommon to perform CT-guided biopsies of soft tissue sarcomas, primarily due to the real-time information required to avoid complications involving important neurovascular structures; thus, it is challenging to compare the diagnostic accuracy of CT-guided biopsies for soft tissue sarcomas [15].

A common use of US-guided biopsies is in the sampling of suspicious lymph nodes. A retrospective analysis performed by Wilczynski et al. [16] examined at 793 cases where US-guided core needle biopsies (US-CNB) were performed. Wilczynski et al. found the diagnostic accuracy to be 95%. Understandably, due to the high diagnostic accuracy, US-guided CNB and US-guided fine needle aspirations (FNA) have replaced the need for surgical lymph node excision. While both US-guided CNB and US-guided FNA are both used for biopsies, it is important to understand the difference in diagnostic accuracy. A meta-analysis performed by Balasubramanian et al. [17] analysed biopsy results from 1353 patients across six studies and found a diagnostic accuracy of 94% for US-guided CNB and 87% for US-guided FNA. The difference in the diagnostic accuracy is largely related to the difference in the size of sample acquired for biopsy. The US-CNB provide a larger, more representative tissue sample suitable for comprehensive histological evaluation, which is critical for distinguishing benign from malignant conditions and for a more accurate diagnosis, whereas FNA provide relatively less.

While CT-guided liver biopsies are performed, US-guided liver biopsies are also commonplace. A study conducted by Wu et al. [18] examined 1030 participants who underwent US-guided biopsies of focal liver lesions and demonstrated a diagnostic accuracy of 93%. Another study performed by Buscarini et al. [19] examining 2091 US-guided fine-needle biopsies to diagnose focal liver lesions demonstrated a diagnostic accuracy of between 93.4% and 95.1%. Thus, it would seem that the diagnostic accuracy lies in the range of 93%-95.1%. By contrast, CT-guided liver biopsy has a diagnostic accuracy ranging from 80% to 95% [1,2]. 

While existing techniques in tissue sampling have proven to be effective and accurate, there has been much research and advancements in biopsy techniques to help ensure high diagnostic accuracy and reduce complications associated with biopsies.

New image-guided techniques

Fusion Imaging

One area of growth in biopsy techniques is the use of fusion imaging in the radiology community. Fusion imaging is a technique that combines multiple imaging modalities to provide a more comprehensive and accurate visualization of a target area. It involves merging images from different modalities, such as US, CT, MRI, or positron emission tomography (PET), to create a fused image that incorporates the strengths of each modality [20,21]. It allows for more specific and sensitive imaging and thus tissues biopsies.

PET/CT Biopsy

A study performed by de Jong et al. [22] on 69 patients who had bone metastases secondary to prostate compared the use of PET/CT-guided biopsies to CT-guided biopsies. The study involved the use of ^68^Ga-prostate-specific membrane antigen (PSMA) as a diagnostic marker for PET scanning of prostate cancer (PCa) metastases. PET/CT is increasingly used in the setting of biochemical recurrence, as it has a high sensitivity and specificity for the early detection of PCa. The authors defined the success rate as: tumour percentage >30%. The success rate of CT-guided bone biopsies for molecular analyses in metastatic PCa patients is approximately only 40%, whereas they found that the success rate of ^68^Ga-PSMA-guided (i.e. PET/CT-guided) biopsies for molecular analyses was 70%. The study indicates that PET/CT-guided fusion biopsy has a higher accuracy than non-fusion-guided techniques. 

*PET/CT US Fusion-Guided Biopsy* 

There has been trimodal fusion of methods involving not just PET/CT but also US when guiding for biopsies. A prospective study by Venkatesan et al. [23] performed 33 PET/CT US-guided biopsies on 25 patients. Targets demonstrated heterogeneous fluorodeoxyglucose (FDG) uptake, were not well seen, or were totally inapparent at conventional imaging. Preprocedural FDG PET scans were rigidly registered through a semiautomatic method to intraprocedural CT scans. Coaxial biopsy needle introducer tips and RF ablation electrode guider needle tips containing electromagnetic sensor coils were spatially tracked through an electromagnetic field generator. Real-time US scans were registered through a fiducial-based method, allowing US scans to be fused with intraprocedural CT and pre-acquired FDG PET scans. A visual display of US/CT image fusion with overlaid co-registered FDG PET targets was used for guidance. Navigation software enabled real-time biopsy needle and needle electrode navigation and feedback. The study demonstrated a diagnostic rate of 86.1%.

US/PET-CT fusion imaging guidance increases accuracy that can be obtained from combining all three modalities, which facilitates biopsies of relatively inconspicuous lesions. Pitfalls of this technique include timing challenges, primarily ensuring that contrast-enhanced CT must be performed immediately after PET in order to facilitate multimodal fusion with real-time US by means of internal anatomical markers. Additionally, the image co-registration process may be time-consuming when different imaging modalities have been acquired in different phases of the respiratory cycle, thereby increasing the overall time required for this type of biopsy method [24].

MRI/CT Fusion-Guided Biopsy

PET/CT-guided biopsies are typically better for lesions that are more difficult to visualize and those that are metastatic. CT imaging has also been fused with MRI to perform accurate biopsies in soft tissues. CT/MRI guidance provides excellent soft tissue contrast and allows for detailed anatomical visualization, which can be particularly useful when targeting lesions in complex or challenging locations [25]. Additionally, MRI can offer functional imaging information and can be valuable for assessing tumour characteristics and staging [26,27]. Donners et al. [7] reviewed 150 CT-guided bone biopsies on 43 patients between 2013 and 2021. The study involved conducting multiparametric bone MRI (mpBMRI), which includes diffusion-weighted and relative fat-fraction images, to identify active bone metastases. These imaging findings were used to select target lesions for CT-guided bone biopsies. Next-generation sequencing (NGS) was then performed on the biopsies. The overall diagnostic accuracy reported was 81% for identifying tumour-positive biopsies, with 84.5% suitable for NGS. By contrast, the study stated the published success rates of CT-guided bone biopsies for tumour diagnosis range between 58% and 90% and between 39% and 82% are suitable for NGS. Overall, the authors state that MRI/CT-guided biopsies have greater accuracy in biopsy sampling, yielding sufficient viable tissue for NGS analysis.

MRI/US Fusion Imaging and Biopsy

**Figure 2:**
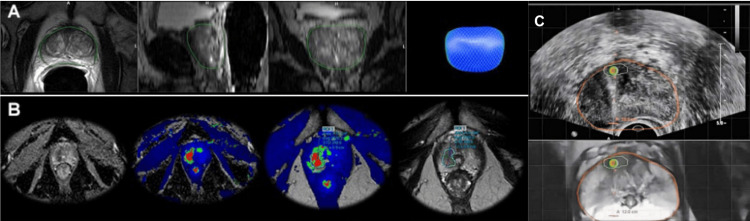
MRI/US-guided prostate biopsy. (A) MRI/US-guided biopsy of the prostate involves initially performing MRI scans of the prostate and rendering a 3D model of the prostate (blue spherical structure), including the location of the suspected tumour using these data. (B) Diffusion-weighted and ADC images can then further elucidate the position of the tumour (coloured green and red). (C) Subsequently, this 3D model, including the tumour’s position, is fused in real time with US imaging, allowing the operator to precisely sample the tumour. The orange line demarcates the outline of the prostate, and the suspected tumour outlined by a white line. Fusing the images allows the operator to accurately pinpoint the needle projection, as shown by the green and orange circles. ADC: apparent diffusion coefficient; MRI: magnetic resonance imaging; US: ultrasound.

In addition to combining MRI with CT, MRI has also been fused with US to facilitate biopsy sampling. Studies have shown the use of this technique in sampling prostate biopsies (Figure 2). A study by Yarlagadda et al. [28] examined 69 patients who underwent MRI/US fusion-guided biopsy. MRI/US fusion-guided prostate biopsy offers equal PCa detection compared with systematic transrectal ultrasonography (TRUS)-guided biopsy, however with significantly fewer tissue cores using the targeted technique (63% less cores sampled, P<0.001). This approach can potentially reduce morbidity in the future if used instead of systematic biopsy without sacrificing the ability to detect PCa, particularly in cases with higher grade disease. Additionally, MRI/US fusion-guided biopsy approach acquires less tissue sampling without compromising diagnostic yield; there is potential for reducing morbidity in the future.

Another study conducted by Kaneko et al. [29] demonstrated that MRI/US-guided biopsies have significantly higher rates of detecting clinically significant PCa than US-guided prostate biopsy technique. MRI/US-guided biopsies have been shown in this study to detect significantly lower rates of clinically insignificant PCa. The dedicated prostate MRI component of this technique can assist in more accurately predicting the Gleason score and provide further information regarding the index cancer location, prostate volume, and clinical stage.

A study conducted by Siddiqui et al. [30], which performed prostate biopsies on 1003 men, demonstrated that targeted MRI/US fusion-guided biopsy is associated with an increased detection of high-risk PCa and a decreased detection of low-risk PCa compared to standard extended-sextant US-guided biopsy. The study found that targeted MRI/US biopsy detected 30% more high-risk PCa cases (Gleason score ≥4 + 3) compared to standard US-guided biopsy. MRI/US-guided biopsy was associated with 17% fewer low-risk PCa cases (Gleason score 3 + 3 or low-volume 3 + 4) compared to standard biopsy. When standard biopsy cores were combined with the targeted approach, an additional 103 cases (22%) of PCa were diagnosed. These additional cases were mostly low-risk (83% low risk, 12% intermediate risk, and 5% high risk). The predictive ability of MRI/US fusion biopsy to differentiate low-risk from intermediate- and high-risk disease was found to be greater than that of standard biopsy or the two approaches combined. The area under the curve (a measure of diagnostic accuracy) was 0.73 for targeted biopsy, 0.59 for standard biopsy, and 0.67 for the combined approaches. In summary, this study demonstrated a more accurate method to sample and differentiate suspicious masses in the prostate compared to the existing monomodal US-guided biopsy technique. 

Molecular Breast Imaging Biopsy

While MRI is an excellent modality for assessing and biopsying soft tissue lesions, there are some disadvantages to using MRI machines. The main disadvantages of using MRI machines include patients experiencing claustrophobia, high machine costs, allergy to contrast, incompatibility with metallic objects, and long scanning times [31].

A recently proposed alternative image-guided modality for breast biopsies is the use of molecular breast imaging (MBI). MBI is a nuclear medicine technique that uses a specialized gamma camera to detect the uptake of radiotracers, such as Tc-99m sestamibi, in metabolically active breast tissue [32]. MBI is particularly useful in cases where mammography may be less effective, such as in women with dense breast tissue [33]. MBI has shown promise in detecting small breast tumours, including those less than 2 cm in diameter. It offers advantages in terms of costs and patient comfort compared to other imaging modalities such as MRI.

A study by Adrada et al. [34] found that overall it suggests that MBI can provide results comparable to MRI in terms of sensitivity while offering a higher specificity, making it an attractive option for breast cancer screening and diagnosis. A meta-analysis comparing the performance of MBI and breast MRI showed that MBI had a sensitivity to detect breast cancer comparable to MRI (82% vs. 89%), with a higher specificity (82% vs. 39%). Additional studies have shown MBI sensitivities ranging from 81% to 91%. MBI as a supplemental imaging modality to mammography has shown an incremental cancer detection rate between 7.7 and 8.8 and additional cancers per 1000 patients screened, with recall rates ranging from 5.9% to 8.4%. The study further evaluated MBI-guided biopsies and found it required similar timing to MRI-guided biopsies.

The authors pose several advantages to MBI-guided biopsies, such as good patient tolerance of the procedure, lack of contraindications to MBI, no use of intravenous gadolinium, the ability to perform a specimen radiograph, and a low cost relative to an MRI-guided biopsy. The open MBI-guided biopsy design is especially advantageous for claustrophobic patients. MBI-guided biopsy can be performed in the sitting or the decubitus position, facilitating lesion accessibility and improving patient comfort. Unlike MRI-guided breast biopsies, there are no contraindications for patients with implanted metallic devices or renal disease, and there are no weight limits. Additionally, MBI does not use intravenous gadolinium, which is reported to have a long-term deposition in the brain of uncertain clinical significance. 

MBI-guided breast biopsies are cheaper than MRI-guided biopsies. The cost of breast MRI and MRI-guided breast biopsies is, on average, $1000 and $3500, respectively. By contrast, the average cost of an MBI examination is $500, with MBI-guided biopsy costs being about half that of an MRI-guided biopsy. MBI-guided biopsies have the potential to replace MRI-guided biopsies due to the significantly lower cost and patient tolerability.

A disadvantage of MBI-guided biopsies is the increased radiation exposure relative to MRI-guided biopsies. MBI typically involves the use of a small amount of radiotracer, such as Tc-99m sestamibi, which emits gamma radiation. The radiation exposure associated with MBI is generally considered to be low, with effective doses ranging from 1.1 to 2.1 mSv. This dose is comparable to or lower than background radiation levels and is similar to the radiation exposure from mammography and tomosynthesis [35]. It is important to note that MBI is still an evolving technology, and further research is needed to establish its full potential and clinical utility.

Shear Wave Elastography-Guided CNB

**Figure 3:**
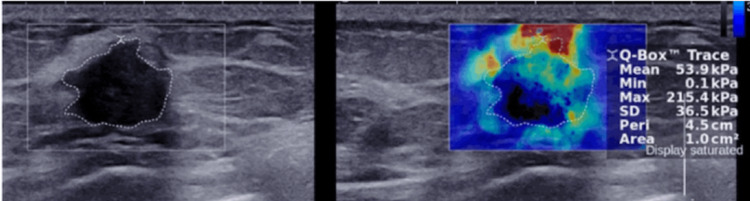
SWE used in demarcating invasive ductal carcinoma in situ. A freehand region of interest was drawn manually around the invasive ductal carcinoma by tracing the border of the mass using SWE to measure lesion elasticity. Stiffer regions are coloured red, with yellow and green indicating intermediate stiffness and blue representing softer tissue. SWE, in this case, can help the operator obtain a biopsy more accurately by targeting the stiffer regions. SWE: shear wave elastography.

Shear wave elastography (SWE)-guided biopsies have been explored with growing interest in order to perform image-guided biopsies. SWE is a recently developed US technique that can visualize and measure tissue elasticity [36]. It works by combining a radiation force induced in a tissue by an ultrasonic beam and an ultrafast imaging sequence capable of capturing the propagation of the resulting shear waves. With SWE, a quantitative measure of the stiffness of a lesion is obtained either in a field of view or pixel by pixel using a colour map. These quantitative measurements of tissue stiffness can improve the accuracy of biopsies (Figure 3) [37]. SWE has been shown to be particularly useful for differentiating malignant and benign breast masses [38,39]. Studies have postulated that SWE in breast biopsies can help reduce unnecessary procedures through decreasing the frequency of unnecessary biopsies performed for benign non-mass lesions [40].

A prospective study performed by Peker et al. [41] examined the impact of SWE guidance on CNB of breast lesions. They conducted the study with 58 patients referred for image-guided CNB of breast lesions larger than 1 cm. In Group 1, 30 lesions were biopsied without SWE guidance and recorded as Biopsy A. In Group 2, 30 lesions were examined with SWE before biopsy, and then two different parts of the lesions were biopsied; biopsies from the relatively rigid areas of the lesions were recorded as Biopsy B, and biopsies from the less rigid areas of the lesions were recorded as Biopsy C. The sensitivity of Biopsy A, Biopsy B, and Biopsy C was 96.7%, 100%, and 100%, respectively. The benign-malignant concordance rates were 94.7%, 100%, and 90% for Biopsies A, B, and C, respectively. The diagnostic concordance rates were 89.5%, 100%, and 90% for Biopsies A, B, and C, respectively. The concordance rate of immunohistochemical subtyping was 100% in Biopsy B and 71.4% in Biopsies A and C. The study concluded that SWE-guided CNB of breast lesions improved sensitivity, diagnostic accuracy, and the accuracy of immunohistochemical subtyping. However, the results indicate that SWE-guided CNB are more accurate for stiffer lesions. 

By contrast, palpation-guided biopsy has a sensitivity of 46.7%, specificity 100%, positive predictive value (PPV) 100%, and a negative predictive value (NPV) of 27.3%. US-guided biopsy was measured to have sensitivity 96.3% and specificity 100%. Overall, it can be ascertained that image-guided biopsy is significantly better than the more rudimentary palpation-guided technique. However, it seems unlikely that SWE-guided biopsies are significantly better than US-guided biopsy. A method which combines both US and SWE poses the benefits of non-invasive differentiation between benign and malignant lesions, particularly stiffer lesions, while also maintaining the potential of real-time tissue sampling [42].

Needle Insertion and Navigation

Image-guided biopsies are practitioner-dependent, like any other technique. It requires sufficient experience and the ability to ascertain the best point of entry and pathway for lesion biopsy. Adequate skill is gained through hours of skill and practice. This opportunity is not always available to all clinicians, as it is highly dependent on suitable teachers and the availability of procedures. As a result, it leads to a large discrepancy in clinicians’ abilities to perform adequate image-guided biopsies. Innovation has focused on reducing that gap through facilitating the passage, pathway, and navigation of needles for biopsies.

One such example is the use of augmented reality (AR) to assist with the biopsy of soft tissue lesions. In a study performed by Bettati et al. [43], a newly developed AR-guided biopsy system was used to perform soft tissue biopsies on phantom lesions. They found an average error of 0.75 cm from the centre of the lesion when AR guidance was used, compared to an error of 1.52 cm from the centre of the lesion during unguided biopsy for soft tissue lesions. The AR-guided system is able to improve the accuracy and could be useful in clinical applications; however, further studies are required to ascertain this.

Another novel method for increasing the accuracy of image-guided biopsies is through the use of “hot needles.” These needles have the ability to determine radioactive isotopes in situ in tumour lesions such as [18F]PSMA-1007 in PCa. Ferraro et al. [44] evaluated the use of these “hot needles” in better detection of tumour lesions in the prostate. In the study, five consecutive patients with suspected PCa underwent [18F]PSMA-1007 PET/CT scans followed by immediate PET/CT-guided and saturation template biopsy. The “hot needles” were used to determine the activity in biopsy cores by measuring counts per minute (cpm) in a gamma spectrometer. Pearson's test was used to correlate counts with histopathology (WHO/ISUP), tumour length, and membranous PSMA expression on immunohistochemistry (IHC). In 43 of 113 needles, PCa was present. The mean cpm was overall significantly higher in needles with PCa (263±396 cpm) compared to needles without PCa (73±44 cpm, P<0.001). In four patients with PCa, the first or second PSMA PET-guided needle was positive for sigPCa with high counts (156-2079 cpm). The use of these “hot needles” could be a helpful way of navigating and sampling biopsies effectively and accurately, lowering the necessity to keep taking further biopsies. This novel technique offers a great deal of potential; however, further larger studies are required to determine the accuracy.

Another method of better discriminating between target lesions and otherwise is to accurately detect differences in electrical conductivity, pH, and glucose concentration between cancerous and normal tissues. The increased tumour metabolism favours anaerobic glycolysis (the Warburg effect) leading to a hypoglycaemic and acidic state, which can be exploited for cancer tissue discrimination. For example, it has been reported that the glucose concentration in the microenvironment of cancerous tissues is 1.2 mM lower than that of normal tissues (1.5-3.3 mM) [45]. One such group looking to exploit these differences is Park et al. [46], who created a 20-μm-thick flexible device with an electrical conductivity sensor, a pH sensor, and a glucose sensor. They showed that their device could measure the parameters in the ranges of human body conditions (conductivity=0.0265 S/m-1.027 S/m, pH=6.6-7.4, and glucose concentration=2 mM-13 mM). Guidance with this device was validated in phantom and animal models, demonstrating the potential for a smart needle able to provide real-time tumour targeting information in vivo to the radiologist.

Needle Biopsy and Robotics

Given the expansion of robotics across most industries over the 21st century, it would naturally follow suit that robotics would make its way into medicine and indeed radiology. Developments have been made in image-guided biopsies to incorporate the use of robots. US-guided biopsy has been the modality of choice to introduce robotic assistance for multiple reasons. US-guided biopsies provide the benefits of having limited setup constraints for robots, being cheap, minimal radiation, and limiting patient discomfort. By contrast, CT-guided biopsy introduces X-ray radiation and requires large expensive machines restricting robot access. While MRI provides high-quality soft tissue images, no radiation, and high spatial resolution, it requires that robotic devices are compatible with the magnetic field and are free of ferromagnetic materials. Thus, US-guided biopsies have become the main contender to couple with robots to enhance biopsy accuracy.

US-guided biopsy robots have the advantage of intraoperative real-time imaging to be used for navigation, but most of the time, the target is defined in preoperative MRI or CT, since a tumour is not always visible in US. This is the most challenging task for a biopsy robot. A typical application is breast biopsies. The breast can be considered a relatively basic structure to perform procedures on, since the structure is isolated from the rest of the body and contains no vital structures. However, the structure is highly deformable, so determining the target location is a challenge. One such workup in which US-guided robotic biopsies work is the following: firstly, the robot acquires volumetric US data of the site; based on these coordinates, the robot performs planning for the intervention. Deformation modelling and tracking during the initial probe positioning are necessary, as the breast is highly deformable. Once the robot is in its final position, the lesion position is updated and the intervention starts. US-guided biopsies are the main diagnostic technique for simple breast lesions found on US. However, not all lesions are detected successfully on US and MRI may be required. As an adjunct to the aforementioned approach, MRI imaging can be incorporated into the workflow and image registration undergone to ensure the lesion is appropriately mapped [47].

Although US-guided biopsies are a strong contender to couple with robots, CT-guided biopsies have been coupled alongside robots too. A study conducted by Maqsood et al. [48] involved lung biopsies on 60 patients who were assigned to two groups: Group A (robot-assisted biopsy) and Group B (conventional CT-guided biopsy). The study found the procedure took on average 25% less time than the conventional CT-guided biopsy. The dose length product, which measures the level of radiation exposure, in the robot-assisted group was on average 32% less than that of the conventional CT-guided biopsy. Overall, only 2% of complications were reported for the robot-assisted group compared to the conventional group. On average, 73% fewer needle adjustments were required compared to the conventional group. The study showed an increased accuracy of needle placement with robotic assistance with <3 mm off target. The study suggested that CT-guided robotic biopsies can be helpful in increasing accuracy, reducing needle adjustments, and reducing procedure time.

Liquid Biopsy

Another novel method being explored is the use of liquid biopsies in identifying cancer early in patients prior to the onset of clinical signs. Liquid biopsies involve detecting circulating tumour DNA (ctDNA), exosomes, or circulating cell-free microRNA. Studies have shown that the use of liquid biopsies can be particularly useful in the detection of pancreatic cancer, which has a more insidious onset. Multiple studies have suggested that through the detection of pancreatic ctDNA, it has become possible to identify early signs of cancer prior to imaging [49-52]. A study by Cohen et al. [53] demonstrated the sensitivity of liquid biopsies in detecting pancreatic cancer ranging from 69% to 98%.

A study on a NGS-based liquid biopsy blood test demonstrated a sensitivity of 54.7% and a specificity of 98.5% [54]. Other studies have demonstrated liquid biopsies to have sensitivities ranging from 38% to 100% in different cancers such as breast, prostate, colorectal, and pancreatic cancer [55,56].

While ctDNA is one of the main components detected in liquid biopsies, studies have shown that 47% of patients with early-stage cancers of any type had detectable ctDNA, whereas the fraction of patients with detectable ctDNA was 82% for patients with advanced cancers [57]. Thus, it is important to note that the accuracy of liquid biopsy is not yet comparable with that of tissue biopsy in cancer confirmation, and its clinical utility is still being examined. Despite this, liquid biopsy has shown promise in aiding early diagnosis, treatment response assessment, and prognosis monitoring across various cancer types, indicating its potential as a complementary method to tissue biopsy.

Discussion

The realm of image-guided biopsies has seen tremendous growth and progress in recent years, offering promising avenues for more accurate, minimally invasive, and patient-friendly diagnostic procedures. The evolution of imaging techniques within CT, US, and MRI-guided biopsies has opened doors for fine-tuning tissue sampling. Current image-guided biopsies involving the aforementioned modalities present many benefits to patients and clinicians alike. Existing methods of biopsy have high diagnostic accuracy but can always perform better, increase comfort to patients, and reduce risks of complications. Both conventional and newer techniques pose their benefits with some drawbacks, thus gaining a holistic perspective, as summarized in Table 1. 

**Table 1:** Summary of conventional and newer techniques for image-guided biopsies providing diagnostic accuracy, benefits of the techniques, and challenges posed with each technique. MBI: molecular breast imaging; SWE: shear wave elastography; CT: computed tomography; MRI: magnetic resonance imaging; PET: positron emission tomography; US: ultrasound.

Technique	Target tissue	Diagnostic accuracy	Common complications/challenges	Source
CT-guided biopsy	Liver	80%-95%	Bleeding, pneumothorax, infection	[1,2]
CT-guided biopsy	Lung	71%-95%	Pneumothorax	[4,5]
CT-guided biopsy	Bone	58%-94%	Wide variation in operator skill	[6-8]
US-guided biopsy	Lung	72%-87%	Effective for peripheral pulmonary lesions, limited in visualizing and accurately sampling small (≤20 mm) or large (≥50 mm) lesions (particularly those with necrosis)	[12]
US-guided biopsy	Soft tissue sarcomas	87%-93%	Tumour seeding, pulmonary embolism	[13,14]
US-guided biopsy	Lymph nodes	94%-95%	Operator-dependent, subsequently can lead to insufficient sampling and a need for surgical excision for a conclusive diagnosis, not suitable for deeper lymph nodes	[16,17]
US-guided biopsy	Liver	88%-95.1%	US can be lower accuracy due to poor lesion visualization (especially compared to CT), operator dependency	[18-20]
PET/CT-guided biopsy	Prostate bone metastases	70%	Higher cost, timing challenges	[23]
PET/CT/US fusion biopsy	Various (neck, axilla, supraclavicular region, chest wall, mediastinum, lung, liver, abdomen, pelvis, and extremities)	86.10%	Timing and image co-registration challenges	[24,25]
MRI/CT fusion biopsy	Bone	81%	Co-registration challenges	[7]
MRI/US fusion biopsy	Prostate	84%	Benefits of lower tissue cores required in fusion biopsy; however, minimal differences noted between MRI/US and US-guided biopsies	[29]
MBI	Breast	82%-91%	Higher radiation exposure than MRI-guided biopsy, insufficient research to justify widespread usage cost advantage over MRI	[35,36]
SWE	Breast	96.7%-100%	Little additional benefits provided over US-guided biopsy; thus, it is unlikely to be in widespread use	[42]
Liquid biopsy	Pancreatic cancer	69%-98%	Less accurate than tissue biopsy, limited research at present	[50-54]

CT guidance excels in deeper tissue biopsies, offering higher accuracy, while US guidance is particularly suited for softer, superficial tissues, such as breast biopsies. CT-guided liver biopsy stands out as a valuable diagnostic tool, demonstrating high accuracy (80%-95%) with major complication rates ranging from 0.6% to 3.2%. The most common complications include bleeding, pneumothorax, and infection. CT-guided lung biopsies exhibit a diagnostic accuracy range of 71%-95%, with the common complication being pneumothorax, found in 17%-26% of cases. In bone biopsies, CT-guided biopsies have a diagnostic accuracy of between 58% and 94%. This wide range highlights the influence of operator skill and lesion characteristics. Typically, CT-guided bone biopsies have higher diagnostic accuracy compared to lung CT-guided biopsies due to the ability to visualize bone lesions in detail and access them accurately for biopsies.

The choice between US-guided and CT-guided lung biopsies is influenced by lesion characteristics and potential complications. US-guided biopsies are preferred for superficial and easily accessible lesions, especially those near the chest wall or in the pleural space. Notably, they exhibit a lower risk of tumour seeding compared to CT-guided biopsies. Diagnostic accuracy for US-guided lung biopsies ranges from 72% to 87%, comparable to CT-guided biopsies, which fall between 71% and 95%. US-guided biopsies are commonly used in other soft tissue biopsies, such as soft tissue sarcomas. The technique’s diagnostic accuracy ranges from 87% to 93% with low complication rates. US-CNB exhibit a high diagnostic accuracy of 95% in sampling suspicious lymph nodes. This accuracy has rendered surgical lymph node excision unnecessary. Differentiating between CNB and FNA, a meta-analysis reveals a diagnostic accuracy of 94% for US-guided CNB and 87% for US-guided FNA. The discrepancy is primarily attributed to the size of the acquired sample, with CNB providing larger and more representative tissue samples.

US-guided liver biopsies are commonplace, with diagnostic accuracy ranging from 88% to 95.1%, making it a reliable choice. Despite having the benefit of contrast to aid the chances of sampling, the overall literature review suggests that CT-guided liver biopsies exhibit a similar diagnostic accuracy to US, ranging from 80% to 95%. The possible driver for this could stem from operator skill and difficulty of lesion site, with CT commonly being used for deeper lesions and US for superficial lesions.

Fusion imaging is a key growth area within image-guided biopsies. The integration of fusion imaging techniques represents a paradigm shift in biopsy procedures, revolutionizing the precision and scope of visualization by combining modalities such as US, CT, MRI, and PET. Key examples of fusion imaging include PET/CT, PET/CT US, and MRI/CT fusion-guided biopsies. The investigation into PET/CT-guided biopsy for patients with bone metastases from PCa yielded compelling results. The success rate of 70% in PET/CT-guided biopsies outperformed conventional CT-guided biopsies (40%). This substantial improvement in success rates underscores the efficacy of PET/CT fusion guidance, particularly in cases involving challenging anatomical conditions. The trimodal fusion approach integrating PET/CT and US demonstrated a diagnostic rate of 86.1%, showcasing its potential in enhancing accuracy, especially for inconspicuous lesions. While the study highlights the success of this fusion technique, challenges related to timing and image co-registration complexity were acknowledged. These challenges, however, may be outweighed by the significant diagnostic gains achieved. Fusion guidance in bone biopsies combining mpBMRI and CT reported a robust diagnostic accuracy of 81%. This surpasses the success rates of conventional CT-guided biopsies (58%-90%), emphasizing the added value of MRI/CT fusion in obtaining viable tissue for NGS analysis. The potential for improved accuracy and precision in targeting lesions, particularly in complex anatomical locations, is a promising aspect of this fusion approach. In the realm of PCa detection, MRI/US fusion-guided biopsies demonstrated equal cancer detection with fewer tissue cores, pointing towards a potential reduction in morbidity. The technique's ability to detect clinically significant PCa positions it as a valuable tool for more nuanced diagnostic decisions. Fusion biopsy presents the potential for higher diagnostic accuracy and better outcomes for patients across anatomical sites. However, it is crucial to acknowledge and address challenges associated with timing and co-registration complexity to ensure the seamless integration of these techniques into routine clinical practice. As fusion imaging continues to evolve, further research and technological refinements will likely solidify its role as a cornerstone in modern biopsy procedures.

MBI-guided biopsies present a compelling alternative to conventional MRI for biopsies. Primary advantages of MBI include enhanced patient comfort, absence of contraindications for patients with metallic devices or renal conditions, and significantly lower costs than MRI. MBI's accuracy has been reported to be similar to MRI, with sensitivity being 82%-91% compared to MRI (89%) in breast cancer detection. The cancer detection rate positions MBI as a valuable supplemental imaging modality alongside mammography. MBI-guided biopsies require similar procedure length as MRI-guided biopsies. Although the effective doses have been reported as comparable to background radiation, it is nevertheless important to acknowledge the drawback of MBI as it exposes patients to greater radiation than MRI. MBI showcases substantial promise in breast cancer diagnostics; however, further studies are required to determine its usage in biopsies as well as in diagnostic imaging.

SWE has emerged as a promising tool for guiding CNB, revolutionizing image-guided biopsy procedures. SWE, a cutting-edge US technique, provides quantitative measures of tissue stiffness, enhancing the accuracy of biopsies. Studies confirm its efficacy in differentiating malignant and benign breast masses, offering the potential to reduce unnecessary procedures by decreasing biopsies for benign non-mass lesions. A prospective study by Peker et al. [41] demonstrated the impact of SWE guidance on CNB for breast lesions, revealing substantial improvements in sensitivity, diagnostic accuracy, and immunohistochemical subtyping accuracy. Biopsies guided by SWE, particularly in relatively rigid areas of lesions, exhibited sensitivity rates of 100%, surpassing conventional palpation-guided biopsies with a sensitivity of 46.7%. Notably, the benign-malignant concordance rates for SWE-guided biopsies were superior to both palpation-guided and US-guided biopsies, emphasizing the potential of SWE in enhancing diagnostic precision. Comparing SWE-guided biopsies to US-guided biopsies, the study suggests that while US-guided biopsies exhibit high sensitivity (96.3%) and specificity (100%), SWE-guided biopsies, especially for stiffer lesions, offer comparable sensitivity (100%) and improved specificity. The combination of US and SWE in a dual-modality approach presents a compelling strategy, providing non-invasive differentiation between benign and malignant lesions, particularly for stiffer lesions. This integrated approach maintains the advantage of real-time tissue sampling while harnessing the strengths of both modalities. Overall, SWE-guided CNB represent a significant advancement, outperforming palpation-guided techniques and demonstrating comparable accuracy to US-guided biopsies. The benefits of SWE are primarily its more specific ability to identify stiffer metastatic lesions. Further research and clinical validation are essential to fully elucidate the potential of SWE in optimizing biopsy outcomes and reducing unnecessary interventions. Overall, however, it seems unlikely that SWE-guided biopsies will replace US-guided biopsies given the existing widespread use and minimal benefits SWE has over US.

The integration of AR into soft tissue biopsies demonstrates promising outcomes. Bettati et al.'s [43] study employing an AR-guided biopsy system showcased a significant improvement in accuracy, with an average error of 0.75 cm compared to 1.52 cm in unguided biopsies. While these findings underscore the potential of AR in enhancing biopsy precision, further studies are warranted for comprehensive clinical validation.

The use of “hot needles” as part of [18F]PSMA-1007 PET/CT-guided biopsy represents an innovative approach to augment the accuracy of image-guided biopsies, specifically in PCa detection. Ferraro et al.'s [44] study demonstrated that these needles significantly improved the accuracy of detecting PCa. This novel technique holds promise for effective and precise biopsy navigation, yet larger studies are imperative to establish its accuracy and widespread utility. Overall, these “smart needles” are innovative methods in cancer detection, which would be more efficient than existing methods; however, further studies are required to demonstrate their safe use and accuracy. Within the realm of innovation lies the incorporation of robotics into image-guided biopsies. Studies involving US-guided robotic biopsies demonstrate enhanced navigation accuracy, particularly in challenging cases like breast biopsies. CT-guided robotic assistance, as demonstrated by Maqsood et al. [48], reduces procedure time, radiation exposure, and complications, emphasizing its potential in increasing accuracy and efficiency. The exploration of liquid biopsies introduces an innovative non-invasive method for early cancer detection through ctDNA, exosomes, or microRNA. Liquid biopsies exhibit notable sensitivity, ranging from 38% to 100% in various cancers, including pancreatic cancer. Despite the current limitations in accuracy compared to tissue biopsy, liquid biopsy holds promise for early diagnosis, treatment response assessment, and prognosis monitoring across diverse cancer types. These innovative methods hold promise as future techniques that can increase diagnostic accuracy, efficiency, and safety for patients.

## Conclusions

The technique used to biopsy a lesion is highly dependent on the tissue itself. The primary agent guiding choice in the developed world is which modality provides the clearest image of the lesion. For bone, we have suggested that CT-guided bone biopsies, while effective, are not as diagnostically accurate as MRI/CT guidance. We have found that MBI provides similar accuracy to US and MRI with no cost savings, added patient safety, or operating advantages, making it an unlikely contender to replace either MRI or US. SWE-guided biopsies may not be ideal for breast biopsies on their own; however, using them in tandem with US to form a SWE/US-guided biopsy would be a helpful method to ensure that stiffer metastatic breast lesions are optimally biopsied. Liver biopsies have primarily been dominated by CT guidance and US and seem likely to remain that way with the potential addition of PET. Across lung biopsies, CT guidance shows superior diagnostic accuracy compared to US. PET/CT fusion imaging seems to be a better option for prostate biopsies, as the research examined offers a much higher diagnostic rate than CT guidance alone. Additionally, the use of MRI/US to perform prostate biopsies provides an option to take fewer tissue cores while also allowing similar accuracy. This can ensure better patient safety. The triple fusion imaging of PET/CT/US provides effective diagnostic rates. Overall, the use of fusion imaging offers a more accurate technique depending on the tissue to biopsy lesions; however, the drawbacks are primarily cost and image co-registration issues. The choice of modality for biopsies ultimately will always somewhat be dictated by costs and availability of machinery, and developing countries are still struggling with the provision of basic machinery. Thus, in the near future, it is likely that we will typically see these more advanced methods in tertiary centres or large hospitals where their efficacy can be evaluated further.
